# Valorization of a Natural Compound Library in Exploring Potential Marburg Virus VP35 Cofactor Inhibitors via an In Silico Drug Discovery Strategy

**DOI:** 10.3390/cimb47070506

**Published:** 2025-07-02

**Authors:** Mohamed Mouadh Messaoui, Mebarka Ouassaf, Nada Anede, Kannan R. R. Rengasamy, Shafi Ullah Khan, Bader Y. Alhatlani

**Affiliations:** 1 Group of Computational and Medicinal Chemistry, LMCE Laboratory, University of Biskra, Biskra 07000, Algeria; mohamedmouadh.messaoui@univ-biskra.dz (M.M.M.); nada.anede@univ-biskra.dz (N.A.); 2 Laboratory of Natural Products and Medicinal Chemistry (LNPMC), Saveetha Medical College and Hospital, Saveetha Institute of Medical and Technical Sciences (SIMATS), Thandalam, Chennai 602105, India; kannan@LNPMC.in; 3 Centre of Excellence for Pharmaceutical Sciences, North-West University, Potchefstroom 2520, South Africa; 4 Inserm U1086 ANTICIPE (Interdisciplinary Research Unit for Cancer Prevention and Treatment), Normandie Université, Universite de Caen Normandie, 14076 Caen, France; shafiullahpharmd@gmail.com; 5 Comprehensive Cancer Center Francois Baclesse, UNICANCER, 14076 Caen, France; 6 Unit of Scientific Research, Applied College, Qassim University, Buraydah 52571, Saudi Arabia

**Keywords:** MARV VP35 polymerase, structure-based virtual screening, molecular docking, pharmacokinetics, drug likeness, DFT, molecular dynamics

## Abstract

This study focuses on exploring potential inhibitors of the Marburg virus interferon inhibitory domain protein (MARV-VP35), which is responsible for immune evasion and immunosuppression during viral manifestation. A combination of in silico techniques was applied, including structure-based pharmacophore virtual screening, molecular docking, absorption, distribution, metabolism, excretion, and toxicity (ADMET) analysis, molecular dynamics (MD), and molecular stability assessment of the identified hits. The docking scores of the 14 selected ligands ranged between −6.88 kcal/mol and −5.28 kcal/mol, the latter being comparable to the control ligand. ADMET and drug likeness evaluation identified Mol_01 and Mol_09 as the most promising candidates, both demonstrating good predicted antiviral activity against viral targets. Density functional theory (DFT) calculations, along with relevant quantum chemical descriptors, correlated well with the docking score hierarchy, and molecular electrostatic potential (MEP) mapping confirmed favorable electronic distributions supporting the docking orientation. Molecular dynamics simulations further validated complex stability, with consistent root mean square deviation (RMSD), root mean square fluctuation (RMSF), and secondary structure element (SSE) profiles. These findings support Mol_01 and Mol_09 as viable candidates for experimental validation.

## 1. Introduction

The *Mononegavirales* order includes the *Filoviridae* family, well-known for causing severe hemorrhagic fevers that can lead to extensive bleeding and organ failure. This family comprises three primary genera: *Ebolavirus, Marburgvirus*, and *Cuevavirus* [1]. The *Marburgvirus* genus consists mainly of two members: Marburg virus (MARV) and its genetic variant, Ravn virus (RAVV). All filoviruses encode seven structural proteins from a 3′ to 5′ negative-sense RNA genome: nucleoprotein (NP), viral proteins VP35 and VP40, glycoprotein (GP), viral proteins VP30 and VP24, and the L protein, which functions as the RNA-dependent RNA polymerase. These viral proteins act as polymerase cofactors and are critical to the viral replication cycle [2,3].

The first epidemic of the Marburg virus (MARV) took place in August 1967, when laboratory workers in Marburg and Frankfurt (Germany) and in Belgrade (Yugoslavia, now Serbia) were confirmed to be infected with a formerly unknown pathogen. The 31 cases (25 initial and 6 subsequent infections) progressed to severe illness, and 7 cases were fatal. One individual who showed symptoms of the disease was diagnosed with the virus retrospectively [4]. In Africa, MARV outbreaks occurred in 1998–2000 in the Democratic Republic of the Congo, with a total of 154 infections and an 83% fatality rate [5]. Furthermore, a major outbreak was reported in Angola during 2004–2005, with 252 cases and a 90% fatality rate [6]. This outbreak served as an international cautionary tale at the time, promoting the development of treatments and drugs to tackle future MARV outbreaks.

The human immune system’s primary response to pathogens is characterized by inflammatory reactions, including the secretion of soluble mediators such as chemokines and cytokines that attract various innate immune cells, including natural killer (NK) cells and dendritic cells, to the site of infection, where they work to eliminate pathogenic entities. Cytokine secretion is particularly prominent during viral infections, and the type of cytokines released, such as interferons (IFNs), often depends on whether the virus is RNA- or DNA-based [7,8].

MARV VP35 polymerase cofactor plays a multifunctional role in the MARV life cycle, making it indispensable for viral replication and immune evasion. Together with VP30 and VP24, VP35 interacts with the nucleoprotein (NP) to encapsidate the viral genome, forming the mature nucleocapsid [7,8]. Beyond its role as a polymerase cofactor, VP35 is crucial in suppressing antiviral immune responses by preferentially binding to viral double-stranded RNA (dsRNA) and weakly capping the ends of MARV dsRNA [9,10], an intermediate product of viral replication. The RIG-I-like receptors (RLRs), including retinoic acid-inducible gene I (RIG-I), melanoma differentiation-associated protein 5 (MDA5), and laboratory of genetics and physiology 2 (LGP2) [11,12], are essential for type I IFN (IFN-α/β) induction through the detection of immunostimulatory RNAs, whether short or long, single- or double-stranded [13,14]. Based on experimental studies by Ramanan et al. [14], MARV VP35 antagonizes the detection of viral dsRNA by RLRs, leading to immune evasion and innate immune system failure [15]. The critical role of VP35 in MARV pathogenesis makes it a promising target for drug discovery. Currently, no FDA- or WHO-approved medications or vaccines exist for Marburg virus infections [16,17]. However, experimental studies have explored several antiviral agents for filoviruses, including Remdesivir [18], Auranofin [19], Apilimod [20], and other macromolecules and antibody treatments [21].

Experimental studies using recombinant Marburg viruses carrying point mutations in the IID of VP35 have demonstrated a loss of the protein’s ability to inhibit host innate immune responses, underscoring the critical role of this domain in immune evasion and viral pathogenesis [22]. Additionally, studies have demonstrated that VP35 proteins from EBOV and MARV interfere with host innate immune signaling, though direct evidence for functional restoration of interferon responses upon VP35 inhibition remains lacking [23,24].

Natural compounds have historically played a significant role in modern medicine, offering a broad spectrum of biological activities. They often exhibit superior efficacy compared to synthetic compounds and generally have lower toxicity, reducing the risk of adverse effects [22,23,24]. The rise of computational approaches has revolutionized drug discovery, providing robust theoretical techniques that yield exceptional results and promising candidate molecules. This study employs in silico methods, including extensive virtual screening of a large natural compound database, molecular docking, pharmacokinetic profiling, and stability approximations using density functional theory (DFT) of the hit molecules and molecular dynamics simulations for systematic stability. The goal is to identify potential hit molecules capable of inhibiting MARV VP35, thus possibly restoring the function of the innate immune system in infected individuals for a potential antagonism of the Marburg virus immune evasion and viral replication mechanism.

## 2. Materials and Methods

### 2.1. Sequence Alignment

To evaluate the degree of sequence conservation between VP35 proteins from Ebola virus (EBOV) and Marburg virus (MARV), a pairwise sequence alignment was performed using the BLASTp tool from the BLAST online platform 2.16.0+/25 June 2024 update available at the NCBI database (https://blast.ncbi.nlm.nih.gov/, accessed on 23 May 2025). The VP35 amino acid sequences were retrieved from UniProt, and the alignment was conducted under default parameters. This analysis aimed to assess the similarity within functional domains, particularly the interferon inhibitory domain (IID), in order to support the comparative interpretation of functional mechanisms between EBOV and MARV.

### 2.2. Protein Preparation

The selected target structure (PDB ID: 4GH9) corresponds to the RNA-binding domain (RBD) of MARV VP35, which is also referred to as the interferon inhibitory domain (IID), as it is functionally responsible for suppressing host innate immune responses through double-stranded RNA (dsRNA) binding and inhibition of interferon signaling. With a high-quality crystal structure (resolution equals 1.65 Å) retrieved from the Protein Data Bank (https://www.rcsb.org/, accessed on 9 January 2024) [25], it provides accurate atomic coordinates and is thus well-suited for computational modeling. All heteroatoms and water molecules were removed, and the structure was optimized using the OPLS3e force field [26], maintaining a convergence threshold of 0.3 Å RMSD for heavy atoms. Protonation states and partial charges of amino acids were adjusted and stabilized under neutral pH conditions using the PROPKA module.

### 2.3. Dataset Preparation and Initial Screening Strategy

The initial compound dataset consisted of the entire January 2022 release of the COCONUT natural products database [27], totaling 407,270 molecules. To optimize the virtual screening workflow prior to the molecular docking stage, a combination of manual and automated filtering steps was applied to handle this large dataset efficiently.

The first selection criterion was molecular weight. A manual filter retained only molecules with masses ranging from 300 to 500 g/mol, reducing the dataset to 204,141 compounds. This range was selected based on established drug likeness principles: molecules with molecular weights below 300 g/mol are often too small to engage effectively with protein targets, whereas those exceeding 500 g/mol typically exhibit poor pharmacokinetic profiles, such as low absorption and permeability, as outlined in Lipinski’s rule of five.

The filtered compounds were then prepared using the LigPrep tool (Schrödinger Release 2020-3: LigPrep, Schrödinger, LLC, New York, NY, USA) within the Maestro 12.5 interface. During preparation, stereoisomer generation was limited to a maximum of 10 per compound. A rotatable bond count (RBC) threshold of ≤10 was applied as an additional filtering criterion to reduce molecular flexibility, which can negatively impact binding affinity and oral bioavailability due to increased entropic penalties. Geometry optimization was performed using the OPLS3e force field. This step yielded a total of 30,599 optimized structures.

To further refine the dataset, the Lipinski rules [28] (“molecular weight less than 500 daltons, number of H-bond donors less than 5, number of H-bond acceptors less than 10 and LogP does not exceed 5”) and Veber rules [29] (“number of rotatable bonds less or equal to 10 with topological polar surface area less than 140 Å”) were systematically applied using the QikProp tool (Schrödinger Release 2020-3: QikProp, Schrödinger, LLC, New York, NY, USA), also within the Maestro 12.5 interface, by only selecting ligands that showed no violations of the Veber and Lipinski rules. This additional screening resulted in 14,773 optimized molecular structures, which were subsequently subjected to e-pharmacophore-based virtual screening. All computational simulations, including ligand preparation, virtual screening, molecular docking, and molecular dynamics, were performed using the licensed Schrödinger Suite 2020-3 and Gaussian 16 W. Access to these tools was granted through the center for high performance computing (CHPC), South Africa, where simulations were executed on high-performance computing clusters equipped with Intel Xeon Gold CPUs and Infinity band networking.

### 2.4. E-Pharmacophore Virtual Screening and Molecular Docking

A pharmacophore is the assembly of a molecule that stores the essential features required for the biological or pharmacological interaction of drugs [30]. Pharmacophores are utilized for screening the compounds that induce a biological response. They are split into two groups: ligand-based and structure-based pharmacophores [31]. Ligand-based pharmacophores require a set of predefined molecular series with proven biological activity [32]. On the other hand, a structure-based pharmacophore, “also called e-pharmacophore”, requires an active ligand that is placed in the target protein active site [33].

For this study, a pharmacophore model was generated using a structure-based manner between the control antiviral agent Remdesivir and the MARV VP35 protein (PDB ID: 4GH9), which contains no co-crystallized ligand. Therefore, the control was docked in using Glide in extra-precision (XP) mode (Schrödinger Release 2020-3: Glide, Schrödinger, LLC, New York, NY, USA) to acquire the necessary protein–ligand for the pharmacophore model generation; the docking site was previously characterized in the literature [34] and comprised the following residues: PRO293, THR291, TYR317, GLN233, LYS237, ILE238, VAL292, VAL286, ILE284, ILE230, VAL234, and PRO282. Resulting a grid box located in the Cartesian coordinates of X = 9.36 Å, Y = 12.99 Å, Z = 2.4 Å, with a cube edge of 20 Å. The output protein–ligand complex was used to generate the e-pharmacophore model with the PHASE module [35] (Schrödinger Suite 2020-3), capturing steric and electronic features essential for molecular recognition.

The previous 14,773 output compounds were screened using the virtually generated e-pharmacophore model. The latter step outputted 2526 natural compounds. Furthermore, using a fitness score criterion greater of equal to 1.0, only 1712 molecules were selected for molecular docking, while considering the same molecular docking conditions from glide docking precision to binding site parameters. After the glide docking step, all ligands with an xp docking score lower than the control were selected for the pharmacokinetic prediction “70 molecules left”.

### 2.5. ADMET, Drug Likeness, and Antiviral Activity Prediction

When developing/researching new drugs, several important factors must be addressed, including physiochemical properties, administration instructions, and even the anatomical status of the patient [36]. Toxicity is very significant in pharmacokinetic studies, as the primary aim is to prevent undesirable effects and phenomena in the human organism by evaluating candidate molecules from various points of view. The protox-3.0 web server (https://tox.charite.de/protox3/, accessed on 24 April 2024) [37] was used to predict toxicity endpoints and the oral toxicity class “14 molecules left”. Additional pharmacokinetic and physicochemical descriptors were maintained with the pkCSM online machine learning platform (https://biosig.lab.uq.edu.au/pkcsm/prediction, accessed on the 1 May 2024) [38], “2 HIT molecules left”.

As for the medicinal chemistry, the latter profiles were approached via SwissADME (http://www.swissadme.ch/, accessed on 1 May 2024) [39] for drug discovery support. Furthermore, PASS (Prediction of activity spectra for substances) prediction (https://www.way2drug.com/antivir/, accessed on 6 May 2024) [40] was used to predict possible inhibition of viral targets.

### 2.6. Chemical Stability and Reactivity

Global quantum chemical (QC) descriptors [41] were calculated after conducting DFT (“density-functional theory”)-based energy minimization in Gaussian 16 W [42] with b3lyp hybrid density functional, including the 6-311G++(d, p) basis set, allowing us to obtain the energies of the main natural bonding orbitals (NBOs), E_HOMO_ (highest occupied molecular orbital energy), and E_LUMO_ (lowest unoccupied molecular orbital energy). The global QC descriptors were calculated according to “Equations (1)–(8)” [43], including energy gap, ΔE; ionization potential, IP; electron affinity, EA; hardness, η; softness, σ; electronegativity, χ; chemical potential, μ; and electrophilicity index, ω:

(1) $$\mathrm{Energy}\;\mathrm{gap}:\;\Delta E=E_{\mathrm{LUMO}}-E_{\mathrm{HOMO}}$$

(2) $$\mathrm{Ionization}\;\mathrm{potential}:\;\mathrm{IP}=-E_{\mathrm{HOMO}}$$

(3) $$\mathrm{Electron}\;\mathrm{affinity}:\;\mathrm{EA}=-E_{\mathrm{LUMO}}$$

(4) $$\mathrm{Hardness}:\;\eta =\frac{\mathrm{IP}-\mathrm{EA}}{2}$$

(5) $$\mathrm{Softness}:\;\sigma =\frac{1}{2\eta }$$

(6) $$\mathrm{Electronegativity}:\;\chi =\frac{\mathrm{IP}+\mathrm{EA}}{2}$$

(7) Chemical potential: μ=−χ

(8) $$\mathrm{Electrophilicity}\;\mathrm{index}:\;\omega =\frac{\mu^{2}}{2\eta }$$

### 2.7. Molecular Dynamics

**Figure 1:**
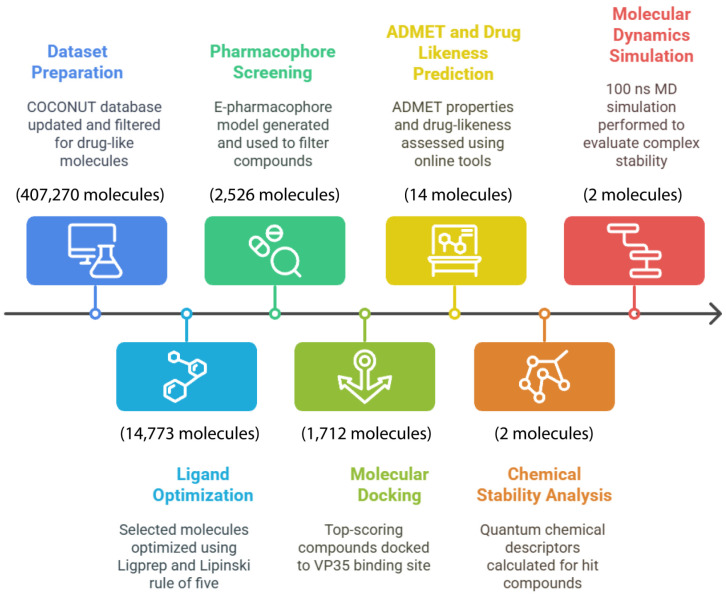
Computational drug discovery workflow for Marburg virus VP35 inhibitors.

Molecular dynamics simulations were performed using the software Desmond 6.31 from Schrödinger LLC in New York, NY, USA. Using the NPT ensemble, we set the temperature to 300 °K and the pressure to 1 bar [44], noting that Desmond supports a single ensemble for system relaxation. The latter parameters were adjusted via the Martyna–Tuckerman–Klein chain coupling method with a 2.0 ps coupling constant for pressure and the Nosé–Hoover chain coupling method for temperature [45]. The simulations employed the OPLS_2005 force field. The particle mesh Ewald (PME) approach was applied to calculate long-distance electrostatic forces with a cutoff radius equal to 9.0 Å for Coulomb interaction [46]. Moreover, the explicit water solvation model of simple point charge (SPC) was chosen for this simulation [47], Na^+^; Cl^−^ counterions were assigned to assure charge neutrality of the system. Non-bonded forces, which updated short-range forces at every step and long-range forces every 3 steps, were computed [48]. The simulation timeline was set to 100 ns, with a 1 ps relaxation time for each ligand depending on the complex of the system under simulation. Data trajectories were recorded every for subsequent analysis. By deploying the Desmond SID (Simulation Interaction Diagram), both the dynamics and interaction profile of the complex were monitored in a continuous manner by evaluating the RMSD (“root mean square deviation”), RMSF (“root mean square fluctuation”), and protein–ligand contact profile during the simulation. All the methodologies employed are summarized in Figure 1.

## 3. Results and Discussion

### 3.1. Sequence Alignment Between MARV VP35 and EBOV VP35 Proteins

A sequence pairwise alignment of the VP35 proteins of Marburg virus (MARV) and Ebola virus (EBOV, strain Zaire 1976) was performed using BLASTp (NCBI). The overall alignment yielded 44% sequence identity and 61% similarity over 126 amino acids, with no gaps and an E-value of 3 × 10^−34^, indicating statistically significant homology. Preserved residues were mainly found in domains of functional relevance, such as the interferon inhibitory domain (IID), thus supporting the extrapolation of EBOV-derived functional insights to MARV; the output results of the alignment are provided in (Table S1, Figure S1).

To complement the sequence-based comparison, a structural overlay was conducted between EBOV and MARV VP35 proteins. As shown in Figure S2, the superposition reveals a consistent α-helical fold across the two proteins, particularly in regions known to contribute to interferon antagonism. This structural conservation reinforces the rationale for applying EBOV-derived mechanistic insights to MARV VP35 function.

### 3.2. E-Pharmacophore Virtual Screening and Molecular Docking

In this study, Remdesivir (GS-5734), an adenosine nucleoside analog known to inhibit the viral RNA-dependent RNA polymerase (RdRp), was used as a reference compound. Its antiviral effect against MARV has been demonstrated through inhibition of the viral polymerase complex, which includes VP35 as a cofactor [49,50]. Notably, Vanmechelen et al. reported a dose-dependent suppression of MARV replication by Remdesivir, with an EC50 value of 149.2 nM. However, it must be clearly stated that no direct evidence supports the binding of Remdesivir to the VP35 interferon inhibitory domain (IID). Therefore, its use in this context is based on a hypothetical extrapolation, assuming a possible but unproven interaction with VP35 IID. This reference was included solely for comparative purposes in docking analysis, and not to imply a confirmed mechanism of inhibition.

The virtual screening procedure, combined with primary in silico predicted toxicity filtration and Veber rule of three, yielded promising outcomes, identifying several potential candidate molecules. The selected ligands demonstrated stable docking scores, ranging from −6.88 kcal/mol to −5.30 kcal/mol, in comparison to the control compound. Notably, Mol_14 exhibited a docking score equal to that of the control (−5.28 kcal/mol). All selected hits were classified within acceptable toxicity categories according to the Globally Harmonized System (GHS) [51], as predicted using the ProTox-3.0 webserver.

ProTox-3.0 predictions include estimated oral LD_50_ values (in mg/kg) and corresponding toxicity classes (1–6), derived from dose–response modeling based on animal toxicity datasets. These LD_50_ values provide insight into the potential acute toxicity of each compound and help estimate safe exposure thresholds. In this study, only compounds predicted to fall within Class IV or higher (LD_50_ > 300 mg/kg) were retained, reflecting a lower likelihood of acute toxicity in vivo. Furthermore, compounds were filtered based on additional predicted endpoints, including hepatotoxicity, carcinogenicity, immunotoxicity, mutagenicity, and cytotoxicity. Any molecule showing a positive prediction for these endpoints was excluded from further consideration. The final 14 hit molecules passed all filters and are summarized in Table S2.

**Figure 2:**
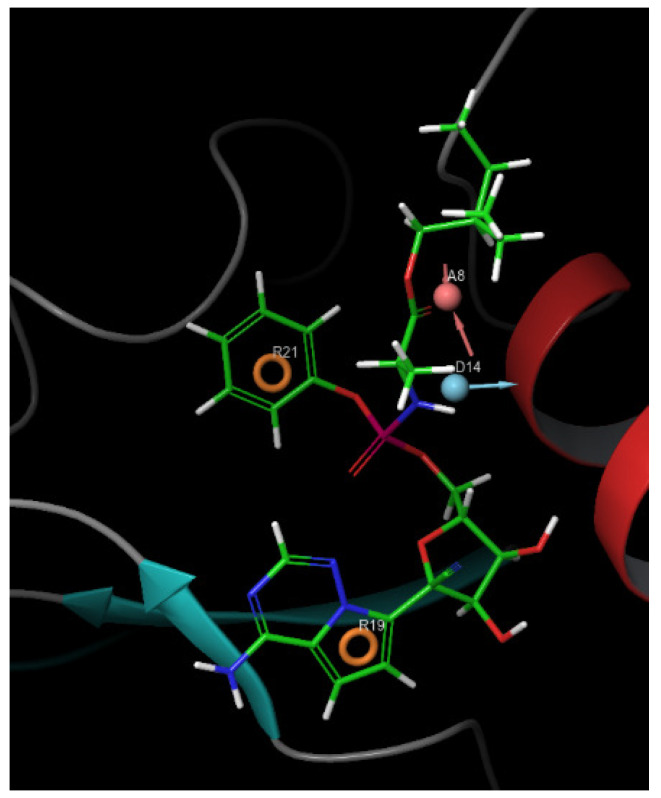
E-pharmacophore model of the Remdesivir–VP35 (PDB ID: 4GH9) complex. The generated pharmacophore includes **two aromatic ring features** (orange rings), **one hydrogen bond donor** (blue sphere), **and one hydrogen bond acceptor** (red sphere).

It was evident that all 14 hit ligands displayed a range of fitness values between 1.01 and 1.49, matching the e-pharmacophore model illustrated in Figure 2. This variation is attributed to the diversity of the model’s features, which comprise a total of four sites: two aromatic rings, one hydrogen donor, and one hydrogen acceptor site.

Moreover, the control compound exhibited a less favorable predicted toxicity profile, with an LD_50_ value of 1000 mg/kg, placing it in toxicity Class IV according to the GHS classification. In contrast, several of the screened ligands demonstrated markedly safer toxicity profiles, with LD_50_ values corresponding to Class V (2000 < LD_50_ ≤ 5000 mg/kg). Notably, Mol_10 exhibited an exceptional safety prediction, classified under Class VI with an LD_50_ of 6800 mg/kg. These improved safety margins can be attributed to the natural origin of the screened compounds, the stringent multi-criteria filtering process, which included drug likeness rules, and the structure-based selection of an appropriate control ligand to guide e-pharmacophore model generation. While exact ED_50_ values are not available, the docking scores obtained suggest potential binding efficiency at low ligand concentrations, supporting the possibility of a favorable therapeutic index when combined with high predicted LD_50_ thresholds.

### 3.3. Pharmacokinetics and Toxicity Prediction

The initial toxicity filtration served as a crucial step, particularly because the MARV VP35 protein facilitates immune evasion by binding to dsRNA, shielding it from detection by the innate immune system [25]. This mechanism leads to immune cell attacks and subsequent immune system breakdown, contributing to the hemorrhagic effects observed in MARV infections. Therefore, any potential toxicity, especially immuno- and hepatotoxicity, was deemed unacceptable. This necessity underpinned the pre-filtration process using the Protox-3 server to enhance the pharmacokinetic evaluation, ensuring the identification of potent antiviral candidates in silico.

The ADMET evaluation, conducted using the pkCSM machine learning platform, covered a comprehensive range of pharmacokinetic properties to support the selection of viable hit molecules. Starting with absorption, which is the initial stage in a drug’s journey through the human body, the results revealed varied characteristics among the 14 candidate hit ligands, as detailed in Table S3. Mol_14 demonstrated the highest water solubility according to the pkCSM model, followed by the control. The remaining molecules exhibited a range of solubility values, influenced by their polarity. Specifically, Mol_04, Mol_10, Mol_12, and Mol_14 displayed water solubility levels comparable to the control, with Mol_14 surpassing the control and other molecules in solubility. Conversely, Mol_07 and Mol_08 were the least soluble, while the rest of the compounds were classified as moderately water-soluble. Regarding Caco-2 cell permeability, a measure of intestinal absorption, Mol_3 exhibited the highest permeability (1.361 × 10^−6^ cm/s), followed by Mol_06 (0.758 × 10^−6^ cm/s), both surpassing the control’s permeability (0.635 × 10^−6^ cm/s). Although Mol_01, Mol_05, and Mol_09 showed below-average intestinal absorption, all compounds exceeded the 30% threshold, indicating likely absorption in the intestinal tract based on the pkCSM model.

All candidate hit compounds, including the control, were predicted to be potential P-glycoprotein (PGP) substrates, except for Mol_11. Additionally, Mol_02, Mol_05, Mol_06, Mol_07, Mol_09, Mol_11, and Mol_13, along with the control, were predicted to inhibit PGP I. Furthermore, Mol_02, Mol_07, and Mol_11 were identified as potential inhibitors of PGP II receptors (Table S3).

In terms of distribution, only Mol_02 and Mol_10 exhibited higher steady-state volume of distribution (VDss) values than the control, suggesting greater plasma concentration. The fraction unbound (Fu), a critical parameter indicating the equilibrium between bound and unbound drug states, further highlighted the pharmacological potential of the hit molecules. The control exhibited strong protein binding (Fu = 0.005), while all proposed ligands, except Mol_07 (with no unbound fraction), demonstrated higher unbound fractions. Notably, Mol_10, Mol_14, and Mol_09 achieved the highest unbound fractions at 0.384, 0.251, and 0.221, respectively (Table S4). Concerning blood–brain barrier (BBB) and central nervous system (CNS) permeability, none of the molecules, including the control, showed significant potential to penetrate the BBB or access the CNS, indicating minimal risk of central neural system distribution [52]. Interestingly, the control exhibited the lowest descriptors for CNS penetration, further confirming its limited neural distribution compared to the candidate molecules.

The metabolism predictions outlined in Table S4 indicated that Mol_01, Mol_05, Mol_06, Mol_09, Mol_10, Mol_13, and Mol_14 are unlikely to exhibit interactive or inhibitory behavior with cytochrome P450 isomorphs. In contrast, the remaining molecules, including the control, were predicted to potentially interfere metabolically as substrates and/or inhibitors of the cytochrome P450 isomorphs, according to the predictive model used in the PKSCM web server. The elimination and toxicity results, detailed in Table S5, revealed noteworthy findings. Total clearance predictions showed that Mol_01, Mol_03, and Mol_13 achieved the highest clearance rates at 1.304, 1.495, and 1.236 log mL/min/kg, respectively. Notably, all ligands outperformed the control in clearance rates, except for Mol_08, which exhibited a lower clearance value of −0.083 log mL/min/kg. Furthermore, the entire series showed negative indications for renal OCT2 substrate potential.

The maximum tolerated dose predictions in the human model highlighted the superiority of most candidate molecules over the control, with the exceptions of Mol_01, Mol_02, Mol_06, Mol_10, and Mol_13. In the oral rat acute toxicity model, significant differences were observed in the predicted half-lethal doses between the candidate molecules and the control. Mol_03 indicated a potential for AMES toxicity, while human ether-a-go-go gene class 2 (hERG II) inhibition was likely associated with Mol_02 through Mol_07, Mol_11, and Mol_12, as well as the control.

### 3.4. Drug Likeness and Antiviral Activity

Since computer-aided drug discovery and design focus on predicting and proposing bioactive molecules with high potency, ensuring their suitability and stability during administration while providing therapeutic activity, various rules and criteria have been established under the term “drug likeness”. Foundational experimental models, including QSAR-related methods, have been pivotal for improving bioavailability. Key drug likeness rules have emerged in the pharmaceutical industry, including the well-known “Rule of Five” by Lipinski and colleagues [28] and the “Rule of Three” proposed by Veber [29].

In this study, SwissADME was utilized to enhance the prediction of the oral bioavailability of the current hits by integrating Veber and Lipinski rules. The ligand preprocessing protocol proved successful, as no violations of Veber and Lipinski rules were observed. Additionally, a strong bioavailability score was predicted for all screened natural ligands, with Mol_08 achieving a score of 0.56, while the rest of the Mol series shared a common score of 0.55.

However, some medicinal chemistry alerts were detected. Pan-assay interference compounds (PAINS) [53] and Brenk alerts [54], which are linked to false positives in therapeutic activity screening [55] and toxic metabolites from problematic fragments [56], respectively, were identified. Specifically, Mol_07 and Mol_11 exhibited both PAINS and Brenk violations, while Mol_08 and Mol_13 and the control showed singular Brenk alerts.

Synthetic accessibility scores were within optimal ranges for all Mol ligands, significantly lower than the control score of approximately 6.43. This indicates that synthesis difficulty is proportional to the synthetic accessibility score [57,58], meaning that higher scores correspond to more challenging synthesis processes. The results of drug-likeness and medicinal chemistry analyses are summarized in Table 1.

To ensure optimal safety and minimize risk with consideration of XP docking score, two CNP natural compounds, Mol_01 and Mol_09, were selected for subsequent computational studies. These selections were justified by their favorable toxicity profiles, pharmacokinetic properties, drug likeness, and medicinal chemistry criteria, with no indications of potential intoxications or violations of small drug design criteria and docking score privilege.

Additional antiviral activity predictions supported this study by identifying potential viral targets, aiding in determining the preferred mechanisms of action against various viral proteins and strains, as assessed through the PASS-online antiviral activity prediction server. The results are detailed in Table 2. Both candidate ligands demonstrated a range of viral targets related to key stages in the viral life cycle. For instance, Mol_01 and Mol_09 were predicted to target the “Replicase polyprotein 1ab”, involved in viral genetic material transcription and replication [59,60]. Mol_09 was also linked to “DNA polymerases”, essential for viral DNA assembly [61,62]. These findings underscore the relevance of the identified hits as potential bioactive molecules against various viruses, depending on the specific viral mechanisms involved. Notably, VP35 is a multifunctional polyprotein responsible for innate immune system inhibition and antagonism in hemorrhagic fevers [63,64]. Such predictions require future experimental validation assays.

### 3.5. Binding Modes and Interaction Maps

**Figure 3:**
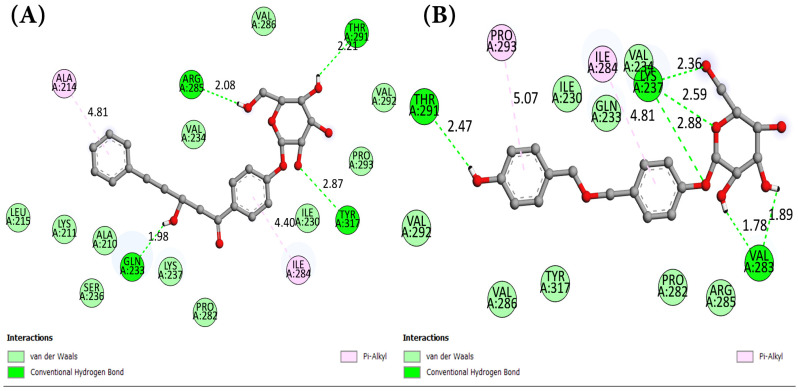
Two-dimensional interaction map scheme with non-covalent interactions: (**A**): VP35-Mol_01 (Com_01); (**B**): VP35-Mol_09 (Com_09).

**Figure 4:**
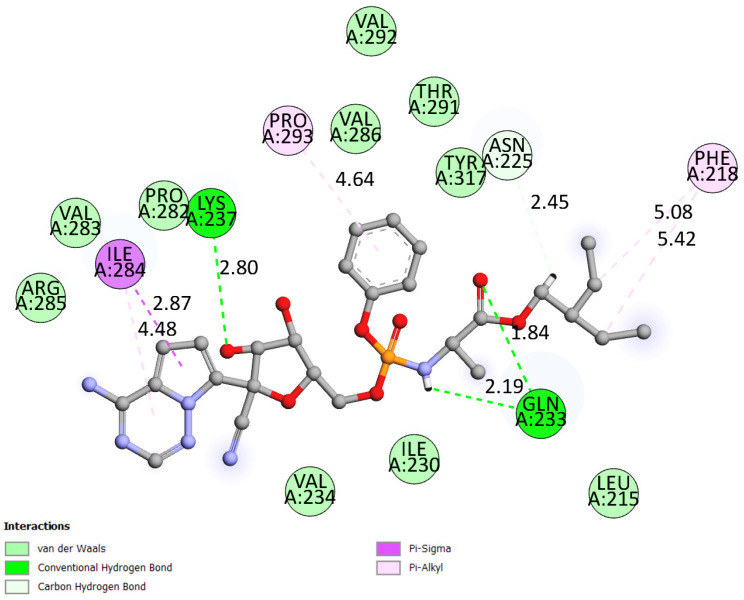
Two-dimensional interaction map of the VP35–Control (Com_ctrl) complex, illustrating key non-covalent binding interactions.

The inclusion of interaction modes was vital for further supporting the conducted computational study. Identifying amino acid residues capable of successful interactions with potential hit molecules could elucidate biological mechanisms of action. After extensive computational virtual screening, ADMET, and drug likeness analyses, interaction maps were generated to better understand the binding modes between each of qualified hit molecules and the target, compared to the control. Remarkable sets of interactions were observed with the candidate hit ligand, characterized by the presence of conventional hydrogen bonds. Com_01 “VP35-Mol_01” was predicted to hold four conventional hydrogen bonds with A:TYR317, A:ARG285, A:THR291, and A:GLN233 (2.87 Å–2.08 Å–2.21 Å–1.98 Å, respectively), along with two pi-alkyl interactions with A:ILE284 (4.40 Å) and A:ALA214 (4.81 Å). On the other hand, Com_09 “VP35-Mol_09” was particularly interesting, with a docking phenomenon with seven conventional hydrogen bonds: A:LYS237 with four bonds (2.88 Å–2.62 Å–2.36 Å–2.56 Å), A:THR291 (2.47 Å), and A:VAL283 with two bonds (1.89 Å–1.78 Å), as well as two pi-alkyl interactions with A:PRO293 (5.07 Å) and A:ILE284 (4.81 Å). The molecular docking interaction data are summarized in Table 3, including the hydrogen bond interactions between selected ligands and key amino acid residues in the VP35 binding pocket. Binding mode surfaces for each complex are visualized in Figure 3 and Figure 4 for 2D interaction mapping in the binding site; Figures S3–S5 highlight the binding sites, hydrogen bond regions, and hydrophobic areas for Mol_01 and Mol_09 and the control agent.

As shown in Table 3, all hydrogen bonds adhered to the maximum length criterion of less than 3.3 Å, consistent with computational study standards [65,66]. Additional hydrophobic interactions were common across complexes Com_01 and Com_09, predominantly featuring pi-alkyl interactions exceeding 4 Å in length. The Com_ctrl “VP35-Control” also exhibited a unique pi-sigma hydrophobic interaction with A:ILE284 (2.87 Å), adding to its distinct interaction profile, along with four pi-alkyl interactions involving A:PHE218 (twice), A:ILE284, and A:PRO293 (5.42 Å, 5.08 Å, 4.48 Å, and 4.64 Å, respectively). As for hydrogen bonds, the control docking outputted three conventional hydrogen bonds inside the binding site with A:GLN233 (1.84 Å), A:LYS237 (2.80 Å), and a second A:GLN233 (2.19 Å), alongside a carbon hydrogen bond with A:ASN225 (2.45 Å).

Mapping of binding site similarities (Figure 3 and Figure 4) revealed overlapping interactions across the ligand–protein complexes, with common involvement of residues such as A:GLN233, A:LYS237, A:ILE284, and A:PRO293. These overlaps support a shared potential mechanism of action, especially concerning RNA mimicry or competitive binding at functionally relevant sites. Furthermore, surface property analysis (Figures S3–S5) indicated that hydrogen bond donor/acceptor surfaces involve THR291 and LYS237—both previously implicated in interferon antagonism—while hydrophobic surfaces favored non-polar interactions with VAL283, ILE284, and PRO293. Notably, the electrostatic surface appeared neutral, suggesting that electrostatic interactions are minimal and do not significantly drive binding affinity in this system.

### 3.6. Density Functional Analysis and Chemical Stability of Candidate HITS

The interpretation of global reactivity in density functional theory (DFT) can be particularly helpful in validating molecular docking results by analyzing chemical interactions and molecular reactive characteristics [67]. The highest occupied molecular orbital (HOMO) and the lowest unoccupied molecular orbital (LUMO) play a crucial role in providing a deep understanding of the electronic characteristics of molecules, especially regarding reactive sites, and enable the assessment of the stability and reactivity of the interactions between ligands and their targets. The ability of a molecule to donate an electron (HOMO) or accept an electron (LUMO) facilitates contact and charge transfer to its enzymatic target [68]. The predicted HOMOs and LUMOs are represented in Figure S6.

Various essential parameters were employed to predict the global reactivity of compounds through HOMO and LUMO energies, including the energy gap (ΔE), ionization potential (IP), electron affinity (EA), electronegativity (χ), chemical potential (μ), chemical hardness (η), softness (σ), and electrophilicity index (ω) [69], as detailed in Table S6.

The difference between the HOMO and LUMO values is represented by the energy gap (ΔE). Generally, a lower ΔE value indicates a more reactive molecule during docking. The reactivity of compounds with the target site can be ranked as Mol_01 (ΔE = 4.54 eV/XP score = −6.88 kcal/mol) > Mol_09 (ΔE = 5.65 eV/XP score = −5.41 kcal/mol). The docking scores of the compounds are confirmed by the ΔE values, indicating that the affinity of Mol_01 for the active site is greater than that of Mol_09.

Two descriptors play pivotal roles in interactions with biological targets. It is known that soft compounds interact more easily with biological targets compared to hard compounds [70]. This means that biological activity increases with softness and decreases with hardness. Compound Mol_01 presents the highest softness value (0.194 eV) compared to Mol_09, and the lowest hardness value (2.27 eV), indicating that Mol_01 may act as a more potent antiviral agent against the targeted enzyme than Mol_09. These results align with the energy gap (Eg) values.

The nucleophilic or electrophilic behavior of molecules is related to their chemical potential values [71]. A large chemical potential and a small electrophilicity index imply nucleophilic behavior, as observed in Mol_09 (ω = 2.16), while Mol_01 (ω = 4.26) exhibits electrophilic behavior. These results are consistent with the HOMO and LUMO energy values; specifically, the HOMO of Mol_09 has the highest value (−6.32 eV), and the LUMO of Mol_01 has the lowest value (−2.13 eV).

The reactivity of molecules can also be validated by their electrophilicity, where a lower value indicates greater chemical reactivity in molecular docking. A similar trend was observed in electronegativity values. However, differences in docking poses can critically influence docking energy rankings. In the cases of Mol_01 and Mol_09, the electrophilicity values did not align perfectly with docking energies, suggesting that the correlation between chemical stability descriptors is only valid when there is high similarity in binding site interactions in terms of quality and quantity, as well as the involved amino acid residues. Nevertheless, the values of chemical stability descriptors remain impactful for validating the molecular stability of candidate hits.

The density of states (DOS) of a material is expressed as the number of distinct states available for electrons to occupy at a certain energy level [72]. The DOS plot presents the distribution of states for each orbital, providing a clear representation of the characteristics of the molecular orbitals, HOMO-LUMO energies, and the energy gap for the studied compounds. The DOS spectrum was generated using GaussSum 3.0 software. The DOS of the two compounds is illustrated in Figure S7. The red and blue bars in the DOS plot represent the HOMO and LUMO levels, respectively. High-intensity DOS at specific energy levels indicates multiple accessible states, while zero intensity suggests no accessible states and no occupation probability.

### 3.7. Molecular Dynamics

**Figure 5:**
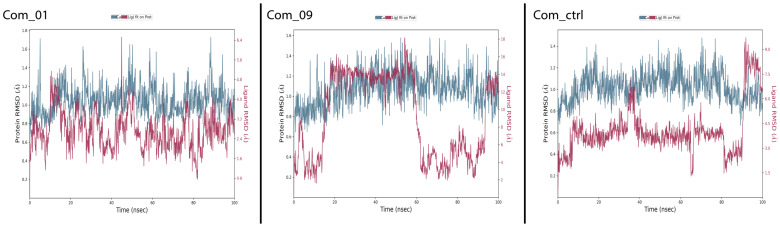
RMSD output plots for the Com_01, Com_09, and Com_ctrl complexes. The blue graph stands for the protein’s C-alpha structural deviation, while the crimson graph stands for the corresponding ligand structural deviation.

Molecular dynamics simulations evaluated structural stability and non-bonded interactions in Com_01, Com_09, and Com_ctrl complexes. Figure 5 shows that Com_01 and Com_09 exhibited favorable C-alpha fluctuation patterns (blue graph) similar to Com_ctrl, indicating that Mol_01 and Mol_09 may induce VP35 conformational changes, potentially disrupting VP35-RBD non-bonded interactions and dissociating viral RNA to reduce MARV VP35-mediated immune evasion. C-alpha RMSD peak values remained within acceptable limits: 1.75 Å (Com_01), 1.6 Å (Com_09), and 1.45 Å (Com_ctrl). Ligand RMSD analysis revealed distinct stability profiles for both complexes. The control ligand (Com_ctrl) remained stable initially but exhibited significant deviation at 38–40 ns, followed by partial stabilization and secondary fluctuations at 90 ns (maximum deviation: 9 Å). The Mol_09 ligand displayed a more complex four-phase behavior: initial deviation increase (0–20 ns), intermediate stabilization with deviations of 9.5–18 Å (20–60 ns), dramatic reduction to 2 Å (post-60 ns), and renewed fluctuation patterns similar to the initial phase. This cyclical behavior indicates potential periodic conformational changes in Mol_09.

Mol_01 in Com_01 showed irregular deviation patterns within 6.4 Å, with structural stabilization occurring after 85 ns, as indicated by reduced fluctuation levels. All complexes (Com_01, Com_09, and Com_ctrl) demonstrated comparable stability with similar C-alpha structural impacts. Com_01 exhibited optimal stability through effective balance of C-alpha and ligand RMSD fluctuations. The results are depicted in Figure 5.

**Figure 6:**
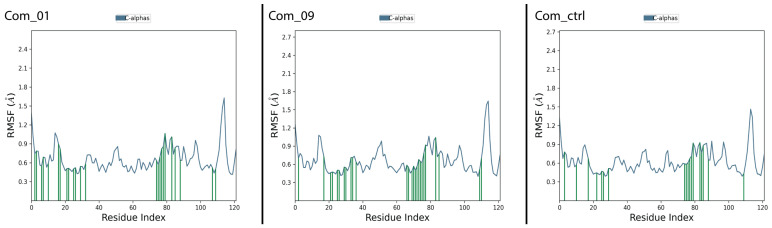
RMSF output plots for Com_01, Com_09, and Com_ctrl. The blue graph stands for the protein’s C-alpha structural fluctuation in function of residue index, and the green lines projected in the residue index axis stand for amino acid residues in contact with the ligand during the MD simulation.

RMSF analysis examined the relationship between amino acid residues involved in non-bonded ligand interactions and their individual fluctuation patterns. A comparison of C-alpha RMSF plots for all complexes and the control in Figure 6 revealed high similarity, supporting mechanistic consistency among systems. Observed differences in fluctuation intensities occurred primarily in residues distant from the binding site, correlating with the RMSD fluctuation patterns previously observed in Figure 5. Green lines on the X-axes indicate amino acid residues in contact with ligands in each complex. Active binding sites showed high similarity across all three complexes, particularly in residue index ranges 0–40 and 60–90. This pattern confirms consistent C-alpha conformational changes during MD simulations across all systems.

**Figure 7:**
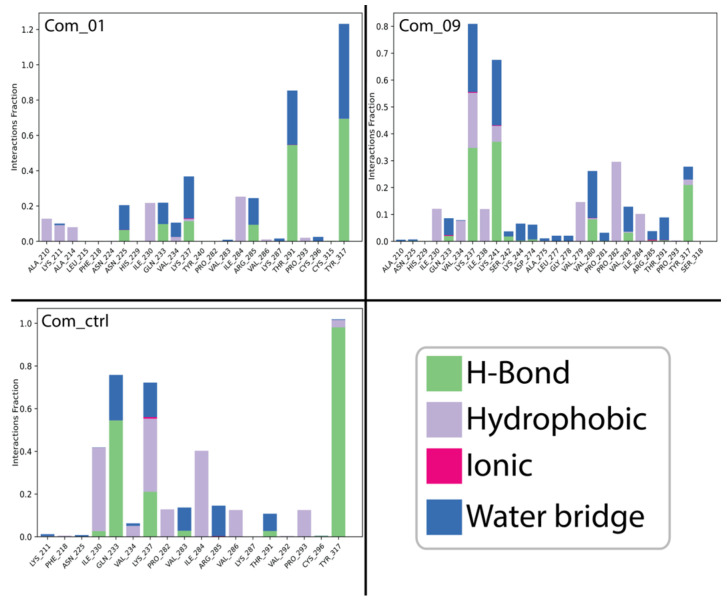
PL interaction fractions as a function of binding site amino acid residues with non-bonded interactions labeled by color in the legend.

The analytical comprehension of the protein–ligand (PL) non-bonded interactions within each complex was crucial for providing a better description. Therefore, the use of the SID interface proved valuable, enabling both quantitative and qualitative PL contact modes to be analyzed. According to the PL interaction analysis shown in the column plots of Figure 7, the Com_01 system predicted a preferential interactive profile between Mol_01 and the residues A:TYR317 and A:THR291, which were characterized by a high fraction of hydrogen bonds and water bridge interactions compared to other amino acid residue contacts.

Com_09 exhibited lower interaction intensities but engaged primarily with residues A:LYS237, A:LYS241, A:VAL280, A:PRO282, and A:TYR317, predominantly through hydrophobic interactions. These interactions likely stabilized C-alpha RMSD fluctuations in Com_09 VP35. Both HITS complexes involved more amino acid residues than Com_ctrl, which showed limited interaction range but higher intensity contributions from A:ILE230, A:GLN233, A:LYS237, A:ILE284, and A:TYR317. These results align with interaction maps (Figure 3 and Figure 4) and binding site data (Table 3), confirming no dissociation occurred during simulations and establishing stability across all complexes.

**Figure 8:**
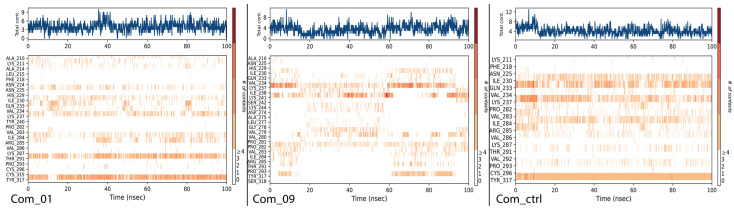
Time-dependent PL contact population for Com_01, Com_09, and Com_ctrl. The upper blue graph stands for total interaction count as a function of simulation timeline; the lower plot stands for the time-dependent contact population for each amino acid residue with the ligand per complex.

Time-dependent protein–ligand contact diagrams (Figure 8) show total interactions throughout the simulation timeline. Upper graphs display interactions in blue, while lower population plots (white to red gradient) indicate bond counts per amino acid residue interacting with the ligand per frame. These results extend the findings in Figure 7, confirming that predominant interaction residues maintained high ligand contact in each complex. Com_01 and Com_ctrl showed steady patterns, with A:TYR317 consistently maintaining 3–4 non-covalent interactions. Total non-bonded interactions ranked Com_ctrl > Com_01 > Com_09. The high interaction density in Com_ctrl correlated with concentrated interaction fractions, a pattern observed across all complexes.

The evolution of the protein structure displayed interesting behavior. According to Figure S8, around 45% to 50% of the protein’s secondary structure elements (SSE) were preserved across the three simulation systems, as shown in the upper three dark blue graphs. The lower graphs highlight the residues involved in SSE changes, including fluctuations, deviations, and possible conformational changes, if any, that occurred during the protein’s interaction with the ligand in the system. The orange color represents alpha-helix secondary structures, while the cyan color indicates beta-sheets.

The similarity in SSE alterations between the Com_01, Com_09, and Com_ctrl complexes revealed that the residues forming the MARV VP35 RNA-binding domain (MARV VP35-RBD), which bridges to the viral nucleic acid, were primarily affected. This domain consists of eight amino acid residues: four are distributed in the same alpha-helix region (ASN 261, GLN 263, THR 267, ARG 271), directly connected to the first beta-sheet in the sequence. The other four residues are located between beta-sheet #2 (SER 299, ARG 301) and the tip of the viral protein (LYS 328, ILE 329) [13,14]. SSE analysis supports the mechanistic similarity between HITS and control complexes through residue index comparison (Figure S8). These computational findings require the experimental validation of Mol_01 “CNP0122944” and Mol_09 “CNP0198479” as potential MARV VP35 inhibitors, including cytotoxicity and pharmacokinetic assessments.

## 4. Conclusions

This study focused on exploring novel antiviral agents to suppress or inhibit the immune evasion protein VP35 of the Marburg virus, a filovirus known for its high fatality and lack of official medication in recent decades. Using modern computational chemistry techniques, a virtual screening process was conducted, beginning with the generation of a structure-based pharmacophore model to screen potential HIT molecules from the extensive COCONUT natural compound database. This was followed by molecular docking, toxicity and pharmacokinetics assessments, molecular stability analyses of the HIT compounds, and molecular dynamics simulations for complex stability. After completing the virtual screening, the molecular docking results identified a set of 14 natural molecules with optimal docking scores, indicating stability compared to the control. ADMET prediction and drug likeness analysis narrowed the candidate ligands down to two potential HITs, with in silico-predicted antiviral activity against viral proteins for possible inhibition. Density functional theory (DFT) analysis was conducted on the top two HIT ligands, Mol_01 and Mol_09, with XP docking scores of −6.88 kcal/mol and −5.41 kcal/mol, respectively, to confirm their chemical stability. Chemical descriptor analysis supported the docking score rankings, with MEP maps indicating a low likelihood of chemical reactivity, confirming their chemical neutrality and favorable orientation for molecular docking. Molecular dynamics simulation results confirmed the stability of all systems, with protein RMSD (Mol_01 below 1.75 Å, Mol_09 below 1.6 Å, and 1.45 Å for the control) and RMSF (highest fluctuation peak found below 1.7 Å for all protein structures per complex) analyses falling within the permitted range. Non-bonded interaction analysis—both qualitative/quantitative and time-dependent (TD) scales—aligned with the binding site analysis from molecular docking. Based on RMSD, RMSF, and TD-PL SSE results, Mol_01 and Mol_09 are likely to share a similar action mechanism to that of the control ligand. Both Mol_01 and Mol_09 are proposed for future in vitro and pharmacokinetic/toxicity studies to further validate their antiviral potency.

## Figures and Tables

**Table 1:** Drug likeness and medicinal chemistry profile of candidate natural ligands and control antiviral.

Tag	Lipinski #Violations	Veber #Violations	B.A Score	PAINS #Alerts	Brenk #Alerts	S.A Score
**Mol_01**	0	0	0.55	0	0	4.88
**Mol_02**	0	0	0.55	0	0	4.2
**Mol_03**	0	0	0.55	0	0	3.05
**Mol_04**	0	0	0.55	0	0	3.15
**Mol_05**	0	0	0.55	0	0	4.58
**Mol_06**	0	0	0.55	0	0	4.2
**Mol_07**	0	0	0.55	1	1	3.83
**Mol_08**	0	0	0.56	0	1	4.45
**Mol_09**	0	0	0.55	0	0	4.68
**Mol_10**	0	0	0.55	0	0	2.8
**Mol_11**	0	0	0.55	1	1	3.9
**Mol_12**	0	0	0.55	0	0	3.21
**Mol_13**	0	0	0.55	0	1	4.84
**Mol_14**	0	0	0.55	0	0	4.17
**Control**	2	2	0.17	0	1	6.43

B.A score: bioavailability score; S.A score: synthetic accessibility score.

**Table 2:** Predicted antiviral targets for CNP candidate hits. * Stands for asymmetric carbon atoms.

Ligand	2D Structure	Target	Confidence
**Mol_01**	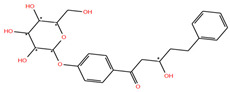	Replicase polyprotein 1ab	0.8015
**Mol_09**	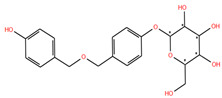	Replicase polyprotein 1ab	0.8733
		DNA polymerase	0.1141
		Human herpesvirus 1 DNA polymerase	0.1141

**Table 3:** Binding modes and interaction outputs of candidate hits and control complex molecular docking.

VP35-Mol_01 “Com_01”			VP35-Mol_09 “Com_09”			VP35-Control “Com_ctrl”		
Residue	Distance	Type	Residue	Distance	Type	Residue	Distance	Type
A:TYR317 A:ARG285 A:THR291 A:GLN233 A:ILE284 A:ALA214	2.87 2.08 2.21 1.98 4.40 4.81	H-bond H-bond H-bond H-bond Pi-Alkyl Pi-Alkyl	A:LYS237 A:LYS237 A:LYS237 A:LYS237 A:THR291 A:VAL283 A:VAL283 A:PRO293 A:ILE284	2.88 2.62 2.36 2.56 2.47 1.89 1.78 5.07 4.81	H-bond H-bond H-bond H-bond H-bond H-bond H-bond Pi-Alkyl Pi-Alkyl	A:GLN233 A:LYS237 A:GLN233 A:ASN225 A:ILE284 A:PHE218 A:PHE218 A:ILE284 A:PRO293	1.84 2.80 2.19 2.45 2.87 5.42 5.08 4.48 4.64	H-bond H-bond H-bond Carbon H-bond Pi-sigma Pi-alkyl Pi-alkyl Pi-alkyl Pi-alkyl

## Data Availability

The original contributions presented in this study are included in the article/supplementary materials. Further inquiries can be directed to the corresponding authors.

## Supplementary Materials

The following supporting information can be downloaded at https://www.mdpi.com/article/10.3390/cimb47070506/s1.

## Abbreviations

The following abbreviations are used in this manuscript:

COCONUT database	Collection of open natural products molecular database
DFT	Density functional theory
dsRNA	Double-stranded ribonucleic acid
EBOV	Ebola virus
FDA	Food and Drug Administration
HOMO	Higher occupied molecular orbital
IFN-1	Interferons type-1
IID	Interferon inhibitory domain
IP	Ionization potential
LD50	Half-lethal dose
LUMO	Lower unoccupied molecular orbitals
MARV	Marburg virus
MD	Molecular dynamics
MDA5	Melanoma differentiation-associated protein-5
PL	Protein–ligand complex
RBD	RNA binding domain
Rdrp	RNA-Dependent RNA Polymerase
RIG-I	Retinoic acid-inducible gene I
RMSD	Root mean square deviation
RMSF	Root mean square fluctuation
SSE	Secondary structure elements
TD	Time-dependent
VP35	Viral polymerase cofactor 35
WHO	World Health Organization

## References

[1] Kuhn J., Andersen K., Bào Y., Bavari S., Becker S., Bennett R., Bergman N., Blinkova O., Bradfute S., Brister J. (2014). Filovirus RefSeq Entries: Evaluation and Selection of Filovirus Type Variants, Type Sequences, and Names. Viruses.

[2] Mühlberger E., Weik M., Volchkov V.E., Klenk H.-D., Becker S. (1999). Comparison of the Transcription and Replication Strategies of Marburg Virus and Ebola Virus by Using Artificial Replication Systems. J. Virol..

[3] Kirchdoerfer R.N., Wasserman H., Amarasinghe G.K., Saphire E.O., Mühlberger E., Hensley L.L., Towner J.S. (2017). Filovirus Structural Biology: The Molecules in the Machine. Marburg- and Ebolaviruses.

[4] Slenczka W., Klenk H.D. (2007). Forty Years of Marburg Virus. J. Infect. Dis..

[5] Bausch D., Nichol S., Muyembe-Tamfum J., Borchert M., Rollin P., Sleurs H., Campbell P., Tshioko F., Roth C., Colebunders R. (2006). International Scientific and Technical Committee for Marburg Hemorrhagic Fever Control in the Democratic Republic of the Congo. Marburg Hemorrhagic Fever Associated with Multiple Genetic Lineages of Virus. N. Engl. J. Med..

[6] Towner J.S., Khristova M.L., Sealy T.K., Vincent M.J., Erickson B.R., Bawiec D.A., Hartman A.L., Comer J.A., Zaki S.R., Ströher U. (2006). Marburgvirus Genomics and Association with a Large Hemorrhagic Fever Outbreak in Angola. J. Virol..

[7] Sanchez A., Kiley M.P., Holloway B.P., Auperin D.D. (1993). Sequence Analysis of the Ebola Virus Genome: Organization, Genetic Elements, and Comparison with the Genome of Marburg Virus. Virus Res..

[8] Bharat T.A.M., Noda T., Riches J.D., Kraehling V., Kolesnikova L., Becker S., Kawaoka Y., Briggs J.A.G. (2012). Structural Dissection of Ebola Virus and Its Assembly Determinants Using Cryo-Electron Tomography. Proc. Natl. Acad. Sci. USA.

[9] Berke I.C., Modis Y. (2012). MDA5 Cooperatively Forms Dimers and ATP-Sensitive Filaments upon Binding Double-Stranded RNA: MDA5 Forms Dimers and Filaments on Binding dsRNA. EMBO J..

[10] Edwards M.R., Liu G., Mire C.E., Sureshchandra S., Luthra P., Yen B., Shabman R.S., Leung D.W., Messaoudi I., Geisbert T.W. (2016). Differential Regulation of Interferon Responses by Ebola and Marburg Virus VP35 Proteins. Cell Rep..

[11] Andrejeva J., Childs K.S., Young D.F., Carlos T.S., Stock N., Goodbourn S., Randall R.E. (2004). The V Proteins of Paramyxoviruses Bind the IFN-Inducible RNA Helicase, Mda-5, and Inhibit Its Activation of the *IFN* -β Promoter. Proc. Natl. Acad. Sci. USA.

[12] Hartmann G. (2017). Nucleic Acid Immunity. Advances in Immunology.

[13] Leung D.W., Ginder N.D., Fulton D.B., Nix J., Basler C.F., Honzatko R.B., Amarasinghe G.K. (2009). Structure of the Ebola VP35 Interferon Inhibitory Domain. Proc. Natl. Acad. Sci. USA.

[14] Ramanan P., Edwards M.R., Shabman R.S., Leung D.W., Endlich-Frazier A.C., Borek D.M., Otwinowski Z., Liu G., Huh J., Basler C.F. (2012). Structural Basis for Marburg Virus VP35–Mediated Immune Evasion Mechanisms. Proc. Natl. Acad. Sci. USA.

[15] Chiang J.J., Davis M.E., Gack M.U. (2014). Regulation of RIG-I-like Receptor Signaling by Host and Viral Proteins. Cytokine Growth Factor Rev..

[16] Health Alert Network (HAN)—00517|First Marburg Virus Disease Outbreak in the Republic of Rwanda. https://emergency.cdc.gov/han/2024/han00517.asp.

[17] Marburg Virus Disease. https://www.who.int/news-room/fact-sheets/detail/marburg-virus-disease.

[18] Warren T.K., Jordan R., Lo M.K., Ray A.S., Mackman R.L., Soloveva V., Siegel D., Perron M., Bannister R., Hui H.C. (2016). Therapeutic Efficacy of the Small Molecule GS-5734 against Ebola Virus in Rhesus Monkeys. Nature.

[19] Abdullah G. (2024). Auranofin Inhibits Ebola Virus Replication by Targeting NP-NP and NP-VP35 Interactions. Ph.D. Thesis.

[20] Vanmechelen B., Stroobants J., Chiu W., Naesens L., Schepers J., Vermeire K., Maes P. (2022). Development and Optimization of Biologically Contained Marburg Virus for High-Throughput Antiviral Screening. Antivir. Res..

[21] Zhang Y., Zhang M., Wu H., Wang X., Zheng H., Feng J., Wang J., Luo L., Xiao H., Qiao C. (2024). A Novel MARV Glycoprotein-Specific Antibody with Potentials of Broad-Spectrum Neutralization to Filovirus. eLife.

[22] Stan D., Enciu A.-M., Mateescu A.L., Ion A.C., Brezeanu A.C., Stan D., Tanase C. (2021). Natural Compounds With Antimicrobial and Antiviral Effect and Nanocarriers Used for Their Transportation. Front. Pharmacol..

[23] Song X. (2024). Antibacterial, Antifungal, and Antiviral Bioactive Compounds from Natural Products. Molecules.

[24] Hu Y., Zhang X., Shan L., Liu L., Chen J. (2024). The Antiviral Activity of Currently Used Medicinal Plants in Aquaculture and Structure–Activity Relationship of Their Active Ingredients. Rev. Aquac..

[25] Bale S., Julien J.-P., Bornholdt Z.A., Kimberlin C.R., Halfmann P., Zandonatti M.A., Kunert J., Kroon G.J.A., Kawaoka Y., MacRae I.J. (2012). Marburg Virus VP35 Can Both Fully Coat the Backbone and Cap the Ends of dsRNA for Interferon Antagonism. PLoS Pathog..

[26] Harder E., Damm W., Maple J., Wu C., Reboul M., Xiang J.Y., Wang L., Lupyan D., Dahlgren M.K., Knight J.L. (2016). OPLS3: A Force Field Providing Broad Coverage of Drug-like Small Molecules and Proteins. J. Chem. Theory Comput..

[27] Sorokina M., Merseburger P., Rajan K., Yirik M.A., Steinbeck C. (2021). COCONUT Online: Collection of Open Natural Products Database. J. Cheminformatics.

[28] Lipinski C.A., Lombardo F., Dominy B.W., Feeney P.J. (2012). Experimental and Computational Approaches to Estimate Solubility and Permeability in Drug Discovery and Development Settings. Adv. Drug Deliv. Rev..

[29] Veber D.F., Johnson S.R., Cheng H.-Y., Smith B.R., Ward K.W., Kopple K.D. (2002). Molecular Properties That Influence the Oral Bioavailability of Drug Candidates. J. Med. Chem..

[30] Pandit N.K., Mann S.S., Mohanty A., Meena S.S. (2023). E-Pharmacophore Modeling and in Silico Study of CD147 Receptor against SARS-CoV-2 Drugs. Genom. Inf..

[31] Yang S.-Y. (2010). Pharmacophore Modeling and Applications in Drug Discovery: Challenges and Recent Advances. Drug Discov. Today.

[32] Kutlushina A., Khakimova A., Madzhidov T., Polishchuk P. (2018). Ligand-Based Pharmacophore Modeling Using Novel 3D Pharmacophore Signatures. Molecules.

[33] Zhou Y., Tang S., Chen T., Niu M.-M. (2019). Structure-Based Pharmacophore Modeling, Virtual Screening, Molecular Docking and Biological Evaluation for Identification of Potential Poly (ADP-Ribose) Polymerase-1 (PARP-1) Inhibitors. Molecules.

[34] Alsaady I.M., Bajrai L.H., Alandijany T.A., Gattan H.S., El-Daly M.M., Altwaim S.A., Alqawas R.T., Dwivedi V.D., Azhar E.I. (2023). Cheminformatics Strategies Unlock Marburg Virus VP35 Inhibitors from Natural Compound Library. Viruses.

[35] Dixon S.L., Smondyrev A.M., Rao S.N. (2006). PHASE: A Novel Approach to Pharmacophore Modeling and 3D Database Searching. Chem. Biol. Drug Des..

[36] Caruba T., Jaccoulet E. (2018). Pharmacologie et Thérapeutiques: Unité d’enseignement 2.11.

[37] Banerjee P., Kemmler E., Dunkel M., Preissner R. (2024). ProTox 3.0: A Webserver for the Prediction of Toxicity of Chemicals. Nucleic Acids Res..

[38] Pires D.E.V., Blundell T.L., Ascher D.B. (2015). pkCSM: Predicting Small-Molecule Pharmacokinetic and Toxicity Properties Using Graph-Based Signatures. J. Med. Chem..

[39] Daina A., Michielin O., Zoete V. (2017). SwissADME: A Free Web Tool to Evaluate Pharmacokinetics, Drug-Likeness and Medicinal Chemistry Friendliness of Small Molecules. Sci. Rep..

[40] Filimonov D.A., Lagunin A.A., Gloriozova T.A., Rudik A.V., Druzhilovskii D.S., Pogodin P.V., Poroikov V.V. (2014). Prediction of the Biological Activity Spectra of Organic Compounds Using the Pass Online Web Resource. Chem. Heterocycl. Compd..

[41] Wang L., Ding J., Pan L., Cao D., Jiang H., Ding X. (2021). Quantum Chemical Descriptors in Quantitative Structure–Activity Relationship Models and Their Applications. Chemom. Intell. Lab. Syst..

[42] Frisch M.J., Trucks G.W., Schlegel H.B., Scuseria G.E., Robb M.A., Cheeseman J.R., Scalmani G., Barone V., Petersson G.A., Nakatsuji H. (2016). Gaussian16 Revision A.03.

[43] Abdou A. (2022). Synthesis, Structural, Molecular Docking, DFT, Vibrational Spectroscopy, HOMO-LUMO, MEP Exploration, Antibacterial and Antifungal Activity of New Fe(III), Co(II) and Ni(II) Hetero-Ligand Complexes. J. Mol. Struct..

[44] (2017). Schrödinger Release 2023: Desmond Molecular Dynamics System MaestroDesmond Interoperability Tools.

[45] Banks J.L., Beard H.S., Cao Y., Cho A.E., Damm W., Farid R., Felts A.K., Halgren T.A., Mainz D.T., Maple J.R. (2005). Integrated Modeling Program, Applied Chemical Theory (IMPACT). J. Comput. Chem..

[46] Toukmaji A.Y., Board J.A. (1996). Ewald Summation Techniques in Perspective: A Survey. Comput. Phys. Commun..

[47] Zielkiewicz J. (2005). Structural Properties of Water: Comparison of the SPC, SPCE, TIP4P, and TIP5P Models of Water. J. Chem. Phys..

[48] Martyna G.J., Klein M.L., Tuckerman M. (1992). Nosé–Hoover Chains: The Canonical Ensemble via Continuous Dynamics. J. Chem. Phys..

[49] Lo M.K., Albariño C.G., Perry J.K., Chang S., Tchesnokov E.P., Guerrero L., Chakrabarti A., Shrivastava-Ranjan P., Chatterjee P., McMullan L.K. (2020). Remdesivir Targets a Structurally Analogous Region of the Ebola Virus and SARS-CoV-2 Polymerases. Proc. Natl. Acad. Sci. USA.

[50] Cross R.W., Bornholdt Z.A., Prasad A.N., Borisevich V., Agans K.N., Deer D.J., Abelson D.M., Kim D.H., Shestowsky W.S., Campbell L.A. (2021). Combination Therapy Protects Macaques against Advanced Marburg Virus Disease. Nat. Commun..

[51] Secretariat U.E. (2023). Globally Harmonized System of Classification and Labelling of Chemicals (GHS).

[52] Muslikh F.A., Kurniawati E., Ma’arif B., Zenmas S.Z., Salmasfattah N., Dhafin A.A., Prasetyawan F. (2023). ADMET Prediction of the Dominant Compound from Mangosteen (*Garcinia mangostana* L.) Using pkCSM: A Computational Approach. Int. J. Contemp. Sci..

[53] Baell J.B., Holloway G.A. (2010). New Substructure Filters for Removal of Pan Assay Interference Compounds (PAINS) from Screening Libraries and for Their Exclusion in Bioassays. J. Med. Chem..

[54] Brenk R., Schipani A., James D., Krasowski A., Gilbert I.H., Frearson J., Wyatt P.G. (2008). Lessons Learnt from Assembling Screening Libraries for Drug Discovery for Neglected Diseases. ChemMedChem.

[55] Yang Z.-Y., Yang Z.-J., He J.-H., Lu A.-P., Liu S., Hou T.-J., Cao D.-S. (2021). Benchmarking the Mechanisms of Frequent Hitters: Limitation of PAINS Alerts. Drug Discov. Today.

[56] Ononamadu C., Ibrahim A. (2021). Molecular Docking and Prediction of ADME/Drug-Likeness Properties of Potentially Active Antidiabetic Compounds Isolated from Aqueous-Methanol Extracts of Gymnema Sylvestre and Combretum Micranthum. BioTechnologia.

[57] Bonnet P. (2012). Is Chemical Synthetic Accessibility Computationally Predictable for Drug and Lead-like Molecules? A Comparative Assessment between Medicinal and Computational Chemists. Eur. J. Med. Chem..

[58] Ertl P., Schuffenhauer A. (2009). Estimation of Synthetic Accessibility Score of Drug-like Molecules Based on Molecular Complexity and Fragment Contributions. J. Cheminformatics.

[59] Denison M.R., Spaan W.J.M., Van Der Meer Y., Gibson C.A., Sims A.C., Prentice E., Lu X.T. (1999). The Putative Helicase of the Coronavirus Mouse Hepatitis Virus Is Processed from the Replicase Gene Polyprotein and Localizes in Complexes That Are Active in Viral RNA Synthesis. J. Virol..

[60] Slanina H., Madhugiri R., Bylapudi G., Schultheiß K., Karl N., Gulyaeva A., Gorbalenya A.E., Linne U., Ziebuhr J. (2021). Coronavirus Replication–Transcription Complex: Vital and Selective NMPylation of a Conserved Site in Nsp9 by the NiRAN-RdRp Subunit. Proc. Natl. Acad. Sci. USA.

[61] Furman P.A., St Clair M.H., Fyfe J.A., Rideout J.L., Keller P.M., Elion G.B. (1979). Inhibition of Herpes Simplex Virus-Induced DNA Polymerase Activity and Viral DNA Replication by 9-(2-Hydroxyethoxymethyl)Guanine and Its Triphosphate. J. Virol..

[62] Gustavsson E., Grünewald K., Elias P., Hällberg B.M. (2024). Dynamics of the Herpes Simplex Virus DNA Polymerase Holoenzyme during DNA Synthesis and Proof-Reading Revealed by Cryo-EM. Nucleic Acids Res..

[63] Leung D.W., Prins K.C., Borek D.M., Farahbakhsh M., Tufariello J.M., Ramanan P., Nix J.C., Helgeson L.A., Otwinowski Z., Honzatko R.B. (2010). Structural Basis for dsRNA Recognition and Interferon Antagonism by Ebola VP35. Nat. Struct. Mol. Biol..

[64] Woolsey C., Borisevich V., Agans K.N., O’Toole R., Fenton K.A., Harrison M.B., Prasad A.N., Deer D.J., Gerardi C., Morrison N. (2023). A Highly Attenuated Panfilovirus VesiculoVax Vaccine Rapidly Protects Nonhuman Primates Against Marburg Virus and 3 Species of Ebola Virus. J. Infect. Dis..

[65] Walker M.G., Mendez C.G., Ho P.S. (2023). Non-classical Non-covalent σ-Hole Interactions in Protein Structure and Function: Concepts for Potential Protein Engineering Applications. Chem. Asian J..

[66] Sagaama A., Issaoui N., Al-Dossary O., Kazachenko A.S., Wojcik M.J. (2021). Non Covalent Interactions and Molecular Docking Studies on Morphine Compound. J. King Saud Univ. Sci..

[67] Vijayaraj R., Subramanian V., Chattaraj P.K. (2009). Comparison of Global Reactivity Descriptors Calculated Using Various Density Functionals: A QSAR Perspective. J. Chem. Theory Comput..

[68] Becke A.D. (1988). Density-Functional Exchange-Energy Approximation with Correct Asymptotic Behavior. Phys. Rev. A.

[69] Eryilmaz S., Gul M., İnkaya E. (2019). Investigation of Global Reactivity Descriptors of Some Perillaldehyde Derivatives in Different Solvents by DFT Method. Indian J. Chem. Technol..

[70] Ismael M., Abdel-Mawgoud A.-M.M., Rabia M.K., Abdou A. (2021). Ni(II) Mixed-Ligand Chelates Based on 2-Hydroxy-1-Naphthaldehyde as Antimicrobial Agents: Synthesis, Characterization, and Molecular Modeling. J. Mol. Liq..

[71] Chaudhary A.P., Bharti S.K., Kumar S., Ved K., Padam K. (2017). Study of Molecular Structure, Chemical Reactivity and First Hyperpolarizability of a Newly Synthesized N-(4-Oxo-2-Phenylquinazolin-3(4H)-Yl)-1H-Indole-2-Carboxamide Using Spectral Analysis. J. Mol. Struct..

[72] Singh H. (2019). A DFT Approach for Theoretical and Experimental Study of Structure, Electronic, Hirshfeld Surface and Spectroscopic Properties of 12-(4-Bromophenyl)-2-(Prop-2-Ynyloxy)-9,10-Dihydro-8H-Benzo[a]Xanthen-11(12H)-on Single Crystal. Chem. Phys..

