# Supplementary material for: Valorization of a Natural Compound Library in Exploring Potential Marburg Virus VP35 Cofactor Inhibitors via an In Silico Drug Discovery Strategy
Source: Curr Issues Mol Biol. 2025 Jul 2;47(7):506. doi: 10.3390/cimb47070506 (PMC12293381; doi:10.3390/cimb47070506)
Supplement: Supplementary file 1 [file cimb-47-00506-s001.zip › cimb-3631501-supplementary.pdf]

Valorization of a Natural Compound Library in Exploring Potential Marburg Virus VP35 Cofactor Inhibitors via an In Silico Drug Discovery Strategy

Mohamed Mouadh Messaoui [1], Mebarka Ouassaf [1,*], Nada Anede [1], Kannan R. R. Rengasamy [2,3], Shafi Ullah Khan [4,5] and Bader Y. Alhatlani [6,*]

-Supplementary Materials-

Table S1. Pairwise sequence alignment statistics between MARV VP35 and EBOV VP35 (Zaire strain) using BLAST.

Protein 1 (Query)	Protein 2 (Subject)	Aligned Length	Identity (%)	Similarity (Positives %)	Gaps (%)	E-value
**MARV VP35**	EBOV VP35 (Zaire 1976)	126	44% (55/126)	61% (78/126)	0%	3e-34

Table S2. Docking scores of screened ligands with the control molecule, considering predicted toxicity classes limited to Class V and beyond (Ligand_IDs from the COCONUT database) comparing to the control with a class IV toxicity.

Tag	Ligand_ID	MW (g/mol)	XP Docking score (kcal/mol)	Fitness (AU)	LD_{50} (mg/kg)	Toxicity class
**Mol_01**	CNP0122944	432.47	-6.88	1.29	3750	V
**Mol_02**	CNP0012461	450.53	-6.40	1.16	3000	V
**Mol_03**	CNP0293495	300.39	-6.24	1.31	2900	V
**Mol_04**	CNP0229626	360.40	-6.16	1.28	2150	V
**Mol_05**	CNP0157910	376.40	-5.72	1.01	3000	V
**Mol_06**	CNP0050562	428.48	-5.56	1.49	3000	V
**Mol_07**	CNP0289655	475.49	-5.49	1.28	4000	V
**Mol_08**	CNP0378444	479.55	-5.45	1.04	2500	V
**Mol_09**	CNP0198479	392.40	-5.41	1.03	3750	V
**Mol_10**	CNP0315754	328.36	-5.40	1.44	6800	VI
**Mol_11**	CNP0405244	496.52	-5.36	1.39	3000	V
**Mol_12**	CNP0218382	360.40	-5.36	1.25	2150	V
**Mol_13**	CNP0207837	432.42	-5.30	1.19	5000	V
**Mol_14**	CNP0155991	392.38	-5.28	1.27	3000	V
**Control**	CID121304016	602.59	-5.28	/	1000	IV

LD_{50} : Half lethal dose – MW: Molecular weight

Table S3. Absorption descriptors report for top 14 ligands and the control predicted *in silico*.

Tag	Water sol (log mol/L)	Caco2 perm (log Papp in 10^{-6} cm/s)	Intestinal absorption (human) (%)	P-gp substrate	P-gp I inhibitor	P-gp II inhibitor
**Mol_01**	-3.985	-0.203	42.113	Yes	No	No
**Mol_02**	-4.042	0.614	79.975	Yes	Yes	Yes
**Mol_03**	-3.816	1.361	90.089	Yes	No	No
**Mol_04**	-3.144	-0.581	54.659	Yes	No	No
**Mol_05**	-3.433	-0.241	41.047	Yes	Yes	No
**Mol_06**	-4.144	0.758	54.437	Yes	Yes	No
**Mol_07**	-4.440	0.353	83.702	Yes	Yes	Yes
**Mol_08**	-4.652	0.626	67.919	Yes	No	No
**Mol_09**	-3.476	-0.184	43.586	Yes	Yes	No
**Mol_10**	-3.035	-0.350	51.392	Yes	No	No
**Mol_11**	-3.831	0.415	89.967	No	Yes	Yes
**Mol_12**	-3.088	-0.433	55.152	Yes	No	No
**Mol_13**	-4.009	0.111	54.971	Yes	Yes	No
**Mol_14**	-2.951	0.382	56.963	Yes	No	No
**Control**	-3.070	0.635	71.109	Yes	Yes	No

Table S4. Distribution and metabolism descriptors report for top 14 ligands and the control predicted *in silico.*

Tag	Distribution				Metabolism						
	VDss (human) (log L/kg)	Fraction unbound	BBB perm (log BB)	CNS perm (log PS)	CYP Substrate		CYP Inhibition				
					2D6	*3A4*	*1A2*	*C19*	*2C9*	*2D6*	*3A4*
**Mol_01**	-0.404	0.134	-1.208	-3.848	No	No	No	No	No	No	No
**Mol_02**	0.510	0.133	-1.319	-2.699	No	Yes	No	Yes	Yes	No	Yes
**Mol_03**	-0.096	0.143	0.011	-2.566	No	No	Yes	Yes	Yes	No	Yes
**Mol_04**	0.020	0.194	-1.437	-3.317	No	No	Yes	No	No	No	No
**Mol_05**	-0.766	0.150	-1.015	-3.812	No	No	No	No	No	No	No
**Mol_06**	-0.350	0.119	-0.731	-3.559	No	No	No	No	No	No	No
**Mol_07**	-0.361	0.000	-1.425	-3.521	No	Yes	No	Yes	Yes	No	Yes
**Mol_08**	-0.981	0.036	-0.584	-2.34	No	Yes	No	Yes	Yes	No	No
**Mol_09**	-0.818	0.211	-1.274	-3.961	No	No	No	No	No	No	No
**Mol_10**	0.433	0.384	-0.898	-2.990	No	No	No	No	No	No	No
**Mol_11**	-0.640	0.073	-1.470	-3.605	No	Yes	Yes	Yes	Yes	Yes	Yes
**Mol_12**	0.046	0.197	-1.435	-3.232	No	No	Yes	No	No	No	No
**Mol_13**	-0.689	0.111	-1.189	-3.888	No	No	No	No	No	No	No
**Mol_14**	-0.880	0.251	-0.955	-3.658	No	No	No	No	No	No	No
**Control**	0.307	0.005	-2.056	-4.675	No	Yes	No	No	No	No	No

Table S5. Elimination and toxicity descriptors report for top 14 ligands and the control predicted *in silico*.

	Elimination		Toxicity				
**Tag**	**Total Clearance (log ml/min/kg)**	**Renal OCT substrate**	**AMES toxicity**	**Max. tolerated dose (human) (log mg/kg/day)**	**hERG I inhibitor**	**hERG II inhibitor**	**Oral Rat Acute Toxicity (LD_{50}) (mol/kg)**
**Mol_01**	1.304	No	No	0.128	No	No	3.310
**Mol_02**	0.821	No	No	-0.001	No	Yes	2.804
**Mol_03**	1.495	No	Yes	0.162	No	Yes	2.169
**Mol_04**	0.484	No	No	0.779	No	Yes	2.384
**Mol_05**	0.870	No	No	0.228	No	Yes	2.793
**Mol_06**	0.581	No	No	-0.079	No	Yes	3.055
**Mol_07**	0.249	No	No	0.533	No	Yes	2.503
**Mol_08**	-0.083	No	No	0.699	No	No	2.195
**Mol_09**	0.869	No	No	0.396	No	No	3.062
**Mol_10**	0.661	No	No	0.088	No	No	2.343
**Mol_11**	0.283	No	No	0.577	No	Yes	2.913
**Mol_12**	0.725	No	No	0.561	No	Yes	2.623
**Mol_13**	1.236	No	No	0.099	No	No	2.925
**Mol_14**	0.351	No	No	0.643	No	No	2.403
**Control**	0.198	No	No	0.150	No	Yes	2.043

Table S6. Chemical stability descriptors of candidate HIT with XP docking scores.

Mol_01				Mol_09			
E_{HOMO}	E_{LUMO}	ΔE	*IP*	E_{HOMO}	E_{LUMO}	ΔE	*IP*
-6.67	-2.13	4.54	6.67	-6.32	-0.67	5.65	6.32
EA	η	σ	χ	*EA*	η	σ	χ
2.27	2.27	0.194	4.4	0.67	2.82	0.125	3.49
μ	ω	*XP docking score*		μ	ω	*XP docking score*	
-4.4	4.26	-6.88		-3.49	2.16	-5.41	

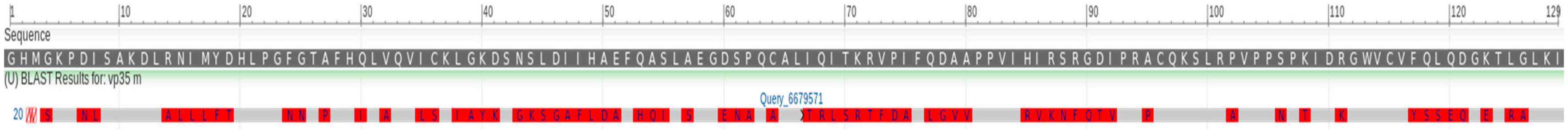

Figure S1. Pairwise sequence alignment between MARV VP35 and EBOV VP35 (Zaire 1976 strain) generated using BLASTp.

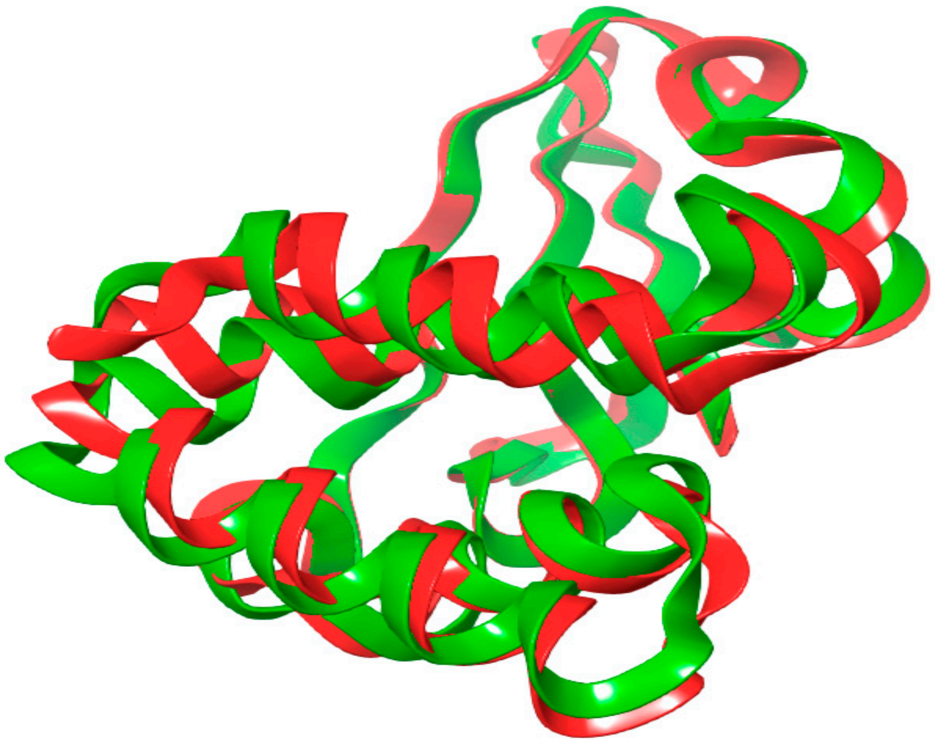

Figure S2. Structural superposition of VP35 proteins from EBOV (Red) and MARV (Green).

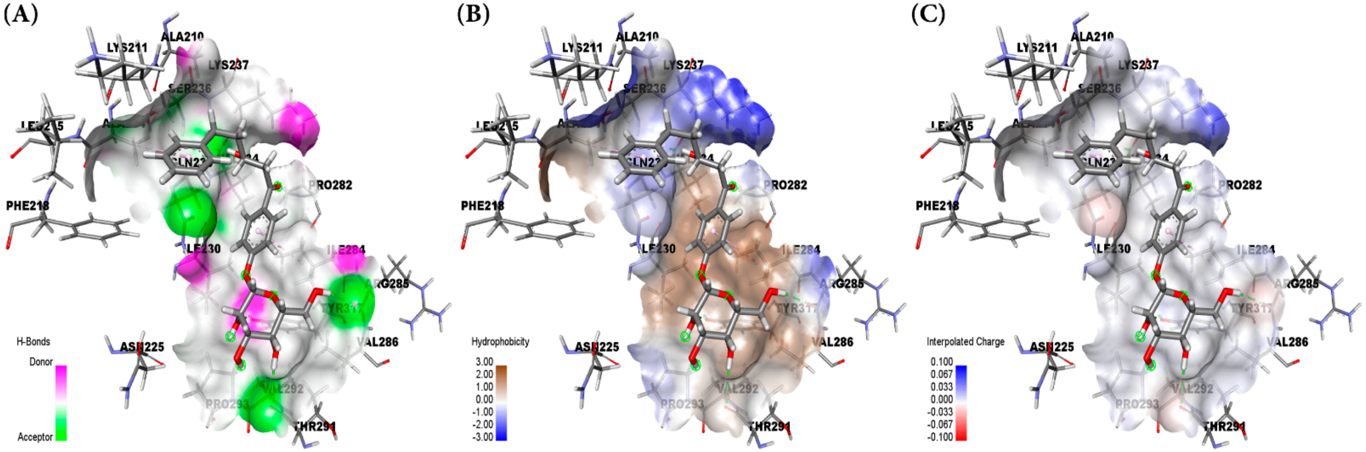

Figure S3. Binding site interactions for Com_01 "VP35-Mol_01", **(A)** H-bonds distribution in the active site cavity surface, **(B)** Hydrophobic mapping in amino acids regions of the binding site, **(C)** Electrostatic mapping in amino acids regions of the binding site.

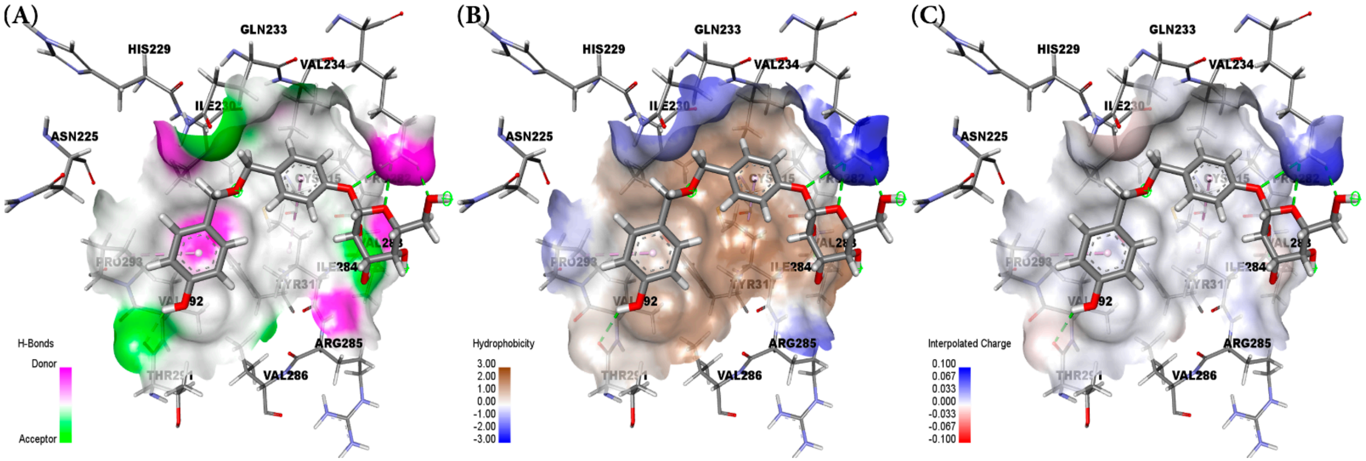

Figure S4. Binding site interactions for Com_09 "VP35-Mol_09", **(A)** H-bonds distribution in the active site cavity surface, **(B)** Hydrophobic mapping in amino acids regions of the binding site, **(C)** Electrostatic mapping in amino acids regions of the binding site.

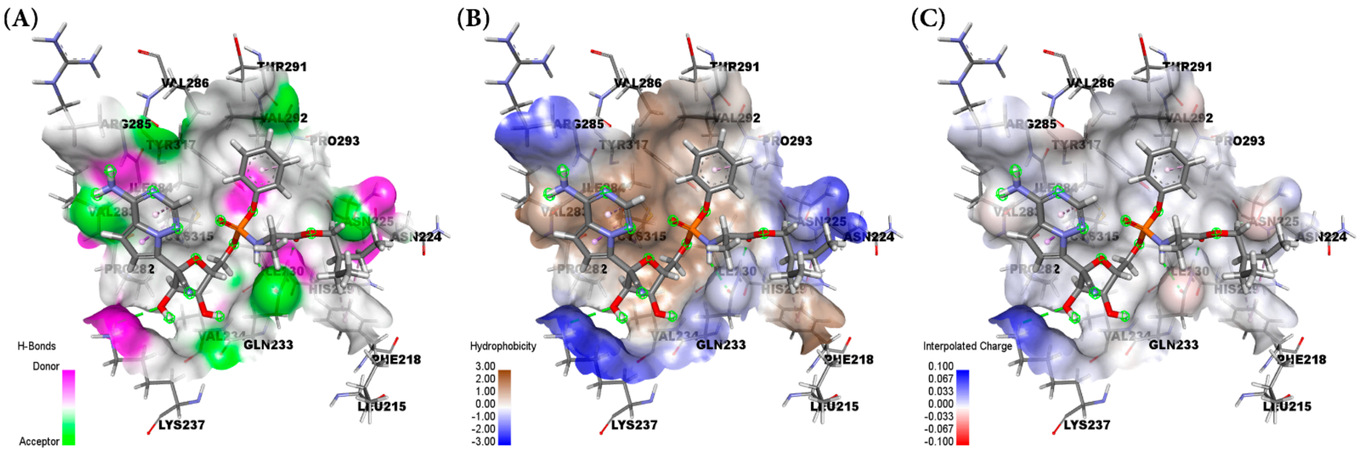

Figure S5. Binding site interactions for Com_ctrl "VP35-ctrl", **(A)** H-bonds distribution in the active site cavity surface, **(B)** Hydrophobic mapping in amino acids regions of the binding site, **(C)** Electrostatic mapping in amino acids regions of the binding site.

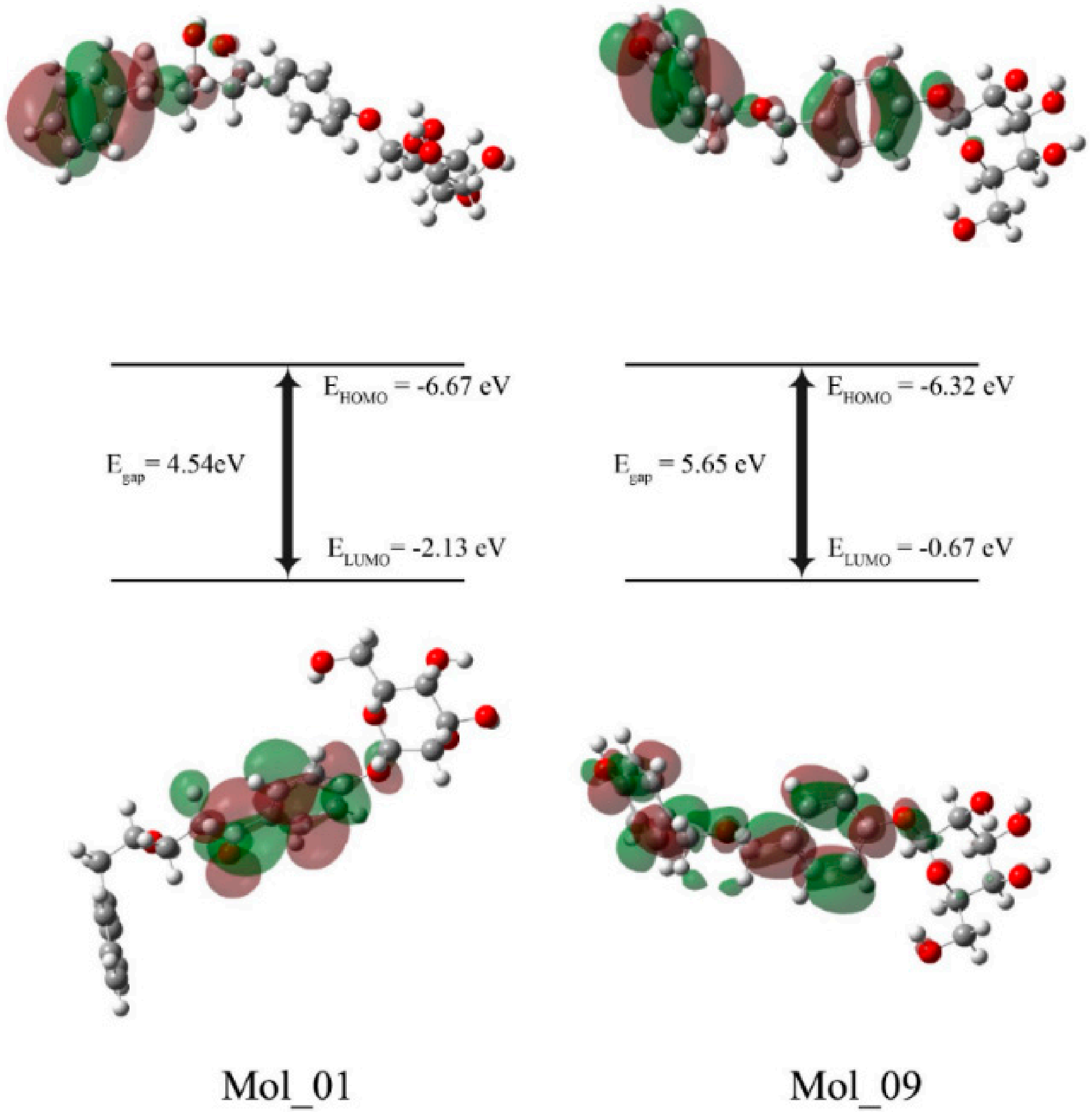

Figure S6. Occupied and unoccupied molecular orbitals including correspondent energies with band gap energy for candidate HITS.

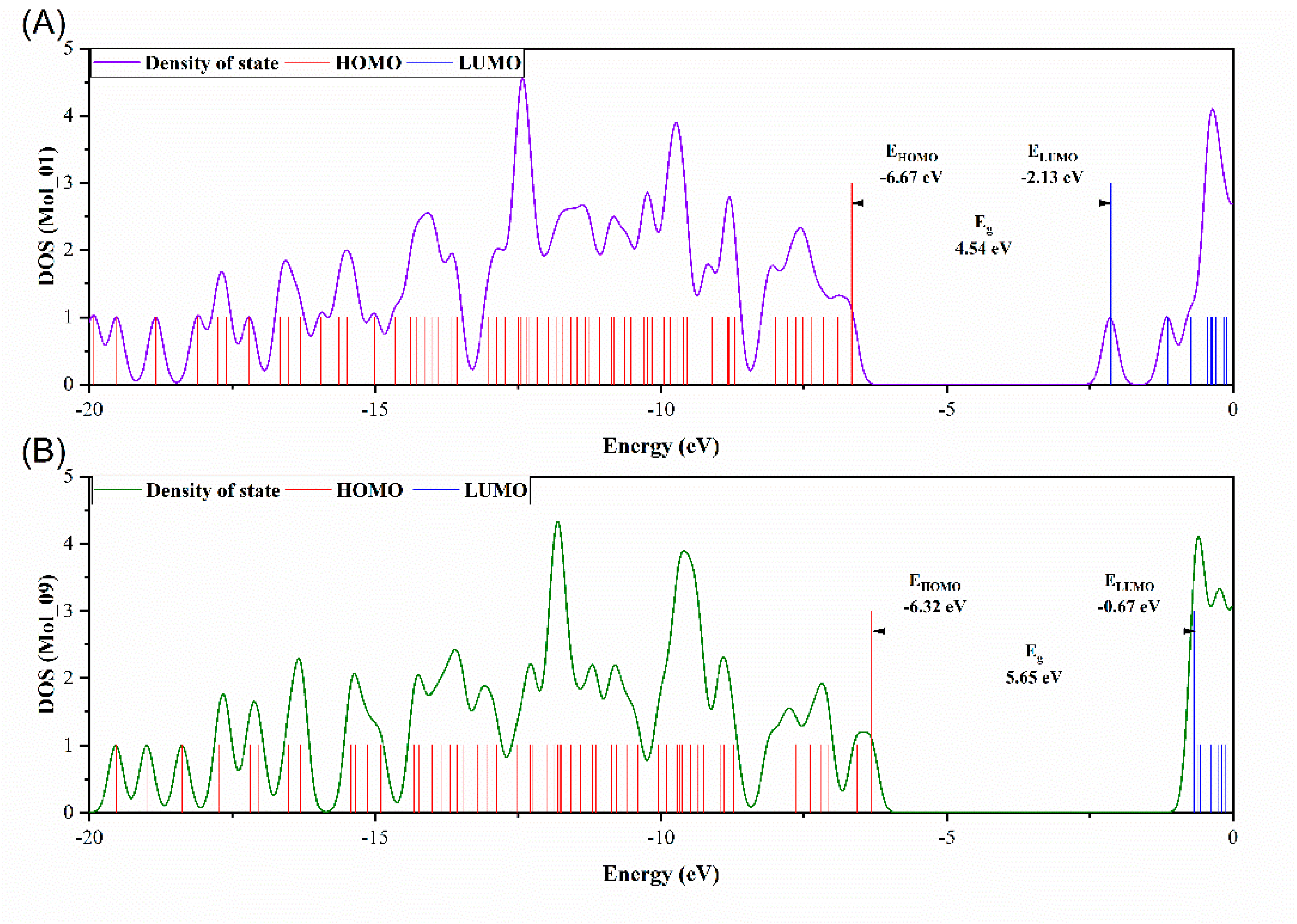

Figure S7. Density of state plots revealing the molecular orbitals energy levels for HOMO and LUMO orbitals for candidate HIT Mol_01**(A)** and candidate HIT Mol_09 **(B).**

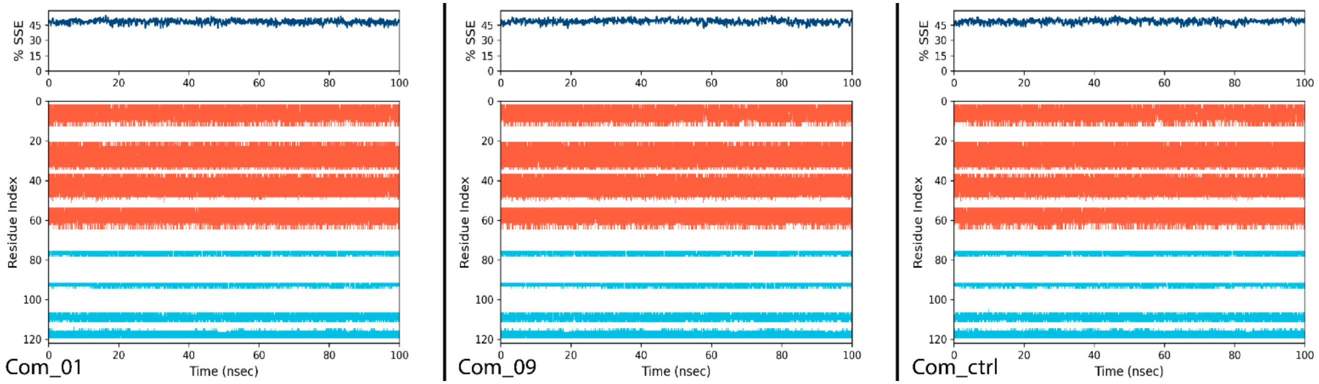

Figure S8. Time-resolved secondary structure elements "SSE" evolution for Com_01, Com_09 and comparing to Com_ctrl. The upper graph expresses affected SSE during the simulation in percentage; the lower plot indicates the affected residues in function of simulation timeline.
